# Complete Genome Sequence of Porcine Deltacoronavirus Isolated in Thailand in 2015

**DOI:** 10.1128/genomeA.00408-16

**Published:** 2016-05-26

**Authors:** Adthakorn Madapong, Kepalee Saeng-chuto, Athip Lorsirigool, Gun Temeeyasen, Anchalee Srijangwad, Thitima Tripipat, Matthew Wegner, Dachrit Nilubol

**Affiliations:** a Department of Veterinary Microbiology, Faculty of Veterinary Science, Chulalongkorn University, Bangkok, Thailand; b Department of Veterinary Pathology, Faculty of Veterinary Science, Chulalongkorn University, Bangkok, Thailand

## Abstract

In Thailand, porcine deltacoronavirus (PDCoV) was first identified in November 2015. The virus was isolated from piglets experiencing diarrhea outbreak. Herein, the full-length genome sequence of the Thai PDCoV isolate P23_15_TT_1115 is reported. The results provide a clearer understanding of the molecular characteristics of PDCoV in Thailand.

## GENOME ANNOUNCEMENT

Porcine deltacoronavirus (PDCoV) is an enveloped, single-stranded, positive-sense RNA virus in the genus *Deltacoronavirus*, family *Coronaviridae* (1–3). PDCoV was first detected in Hong Kong in 2012 (4). The virus was then identified in Ohio, United States, in 2014 and has since been detected in several U.S. states, Canada, South Korea, and China (5–10). Clinical signs of PDCoV infections are similar to, but milder than, porcine epidemic diarrhea virus (PEDV) infections, which belong to the same family (9, 11,12). Recently, PDCoV has been suspected in herds in Thailand. In this study, we report the detection and whole-genome characterization of PDCoV in Thailand.

A surveillance study was conducted to identify the PDCoV presence in Thailand that focused on herds with diarrhea outbreak and low mortality. Six intestinal samples were collected from piglets with diarrhea in five herds experiencing diarrhea outbreak. Total RNA was extracted and detected the presence of PDCoV RNA using primers specific to membrane (M) and nucleocapsid (N) genes. PCR positive samples were further investigated. The full-length genome was sequenced using 16 overlapping regions of each genome, cloned in pGEM-T easy vector (Promega), and sequenced in both directions in triplicate according to the previously reported protocol (9). The 5′ terminal sequences were determined by 5′ rapid amplification of cDNA ends (RACE).

The full-length genome of the Thai PDCoV isolate P23_15_TT_1115 was characterized. The full-length genome sequence of P23_15_TT_1115 is 25,402 nucleotides (nt) in length. Genome organization of the isolate resembles that of other PDCoV genomes with the following gene order: 5′ untranslated region (UTR), open reading frame 1a/1b (ORF 1a/1b), spike (S), envelope (E), membrane (M), nonstructural protein 6 (Nsp6), nucleocapsid (N), nonstructural protein 7 (Nsp7), 3′ UTR. The lengths of ORF 1a/1b, S, E, M, and N genes are 18,786; 3,477; 249; 651; and 1,026 nt, respectively. The phylogenetic tree was constructed based on full-length PDCoV genomes of 23 isolates available in GenBank, and phylogenetic analysis demonstrates that the P23_15_TT_1115 isolate belongs to a group separated from PDCoVs reported in both China and the United States.

The full-length genome of P23_15_TT_1115 was compared to 23 isolates available in GenBank. P23_15_TT_1115 was more highly homologous to PDCoV isolates from China, with nucleotide and amino acid similarities at 97.2 to 97.8% and 93.0 to 94.0%, respectively. In comparison, P23_15_TT_1115 shares a similarity (97.3% and 92.8 to 93.0% at the nucleotide and amino acid levels, respectively) with isolates from the United States. Moreover, the genetic analysis based on the S gene demonstrated that P23_15_TT_1115 is closely related to China PDCoV with similarities of 95.6 to 96.7% and 95.9 to 98.1% at the nucleotide and amino acid levels, respectively. Twenty-four substitutions at the amino acid level were observed between P23_15_TT_1115 and the isolates from China. Moreover, P23_15_TT_1115 owns a deletion of 1 (^51^N) amino acid at position 51, similar to isolates from China.

The results in this study suggest that P23_15_TT_1115 is a novel isolate, closely related to PDCoV isolates from China. The studies investigating the molecular epidemiology, prevalence, and evolution of PDCoV in Thailand are urgently required.

### Nucleotide sequence accession number.

The complete genome sequence of P23_15_TT_1115 has been deposited in GenBank under the accession number KU984334.
